# How Familiarity, Musical Affinity, and ADHD Shape Adolescents’ Perception of Musical Emotions

**DOI:** 10.3390/bs15111570

**Published:** 2025-11-17

**Authors:** Adam Robaczewski, Erika Harkins, Pénélope Pelland-Goulet, Nathalie Gosselin

**Affiliations:** 1 Laboratory on Music, Emotion and Cognition (MUSEC), Department of Psychology, Université de Montréal, Montreal, QC H3C 3J7, Canada; erika.harkins@umontreal.ca (E.H.);; 2 International Laboratory for Brain, Music, and Sound Research (BRAMS), Université de Montréal, Montreal, QC H2V 2S9, Canada; 3 Center for Research on Brain, Language, and Music (CRBLM), Faculty of Medicine, McGill University, Montreal, QC H3G 2A8, Canada; 4 Center for Interdisciplinary Research on Brain and Learning (CIRCA), Université de Montréal, Montreal, QC H3C 3J7, Canada

**Keywords:** musical emotions, familiarity, musical affinity, arousal, emotional valence, ADHD, adolescents

## Abstract

Music serves as a powerful tool for emotion regulation, particularly in adolescents, who experience emotional challenges. Understanding the determinants shaping their perception of musical emotions may help optimize music-based interventions, especially for those with ADHD. This online study examined how familiarity, musical affinity, and ADHD diagnosis influence adolescents’ judgments of musical excerpts in terms of arousal and emotional valence. A total of 138 adolescents (38 ADHD, 100 controls) rated 55 excerpts for arousal, valence, and familiarity using 10-point Likert scales. Musical affinity was conceptualized as a multidimensional construct encompassing musical experience, listening diversity, and receptivity to musical emotions. A cluster analysis identified two affinity profiles (low and high), and ANCOVAs tested the effects of affinity, ADHD, and familiarity on arousal and valence judgments. Familiarity strongly affected both arousal and valence. High-affinity adolescents judged excerpts as more pleasant and familiar, though arousal ratings did not differ between affinity profiles. Familiarity effects on emotional valence were stronger among lower-affinity adolescents. ADHD status did not significantly affect ratings. Overall, the study underscores music’s potential for emotion regulation and its relevance in educational, clinical, and self-care contexts.

## 1. Introduction

Adolescents place music at the core of their daily lives, making them the most frequent consumers compared to other age groups (Bonneville-Roussy et al., 2013; Krause et al., 2015; Lapointe, 2010; Magnan, 2016). Beyond its ubiquity, music constitutes a notable tool for emotional regulation and well-being in everyday life (Baltazar & Saarikallio, 2017; Groarke & Hogan, 2019; Saarikallio & Erkkilä, 2007; Schellenberg & Weiss, 2013). For adolescents, music may therefore provide vital assistance as they navigate emotional challenges typical of this development period, such as identity development and peer acceptance (Tetzner et al., 2017). This role may assume greater importance for adolescents with attention deficit/hyperactivity disorder (ADHD), who often experience emotion dysregulation. It has been demonstrated that this clinical population often encounters difficulties in regulating their emotional state to adapt to environmental demands or to reach one’s goals (Ayano et al., 2023; Graziano & Garcia, 2016). In clinical contexts, music is increasingly used as a therapeutic tool (see Romano et al., 2024 for a review), especially for youth with ADHD, with recent reviews and meta-analyses underscoring potential benefits for mood improvement and emotion regulation (de Oliveira Goes et al., 2025; Martínez-Vérez et al., 2024; Saville et al., 2025). Understanding how adolescents perceive emotions in music is therefore a key aspect in clarifying the potential role of music as a regulatory tool, especially in adolescents with ADHD. As such, the goal of this study was to improve knowledge regarding the ways in which adolescents with and without ADHD perceive musical emotions. To situate the present research, the next sections successively address four central themes: (1) dimensional approaches to musical emotions, (2) the role of tempo and familiarity in musical emotion perception, (3) individual musical affinity variability, and (4) musical emotion perception in ADHD.

### 1.1. Dimensional Approach to Musical Emotions

Over the past few decades, researchers have investigated emotional responses to music using two prominent approaches: categorical and dimensional. The categorical approach, based on general theories of basic emotions (Ekman, 1992), suggests that listeners may recognize and experience different emotions based on musical cues, such as happiness, sadness, or anger (Juslin & Laukka, 2004; Zentner et al., 2008). In the context of musical emotions specifically, however, the categorical approach is limited. Indeed, emotions elicited by music are often complex and hard to confine to discrete categories (e.g., “sad” music eliciting positive feelings; Sachs et al., 2015) and are therefore better conceptualized as spectrums (Cespedes-Guevara & Eerola, 2018; Eerola & Vuoskoski, 2011; Zentner et al., 2008).

As a result, a complementary approach has emerged in research on musical emotions, prioritizing a dimensional framework to allow for nuances and to better reflect the richness and ambiguity of musical experience (Samson & Dellacherie, 2010; Vieillard et al., 2008). Two dimensions of emotions are particularly relevant: arousal (relaxing-stimulating), defined as a physiological or subjective experience of energy or activity, and emotional valence, defined as the pleasant-unpleasant continuum (Eerola & Vuoskoski, 2011; Juslin & Laukka, 2004; Korsmit et al., 2023). These dimensions are widely used in musical emotion research and provide a parsimonious way to assess adolescents’ emotional judgments. In this context, both structural features of music (e.g., tempo) and experiential factors (e.g., familiarity) have been shown to modulate perceived arousal and emotional valence, offering complementary insight into how listeners interpret musical emotions.

### 1.2. The Role of Tempo and Familiarity in Musical Emotion Perception

Among musical parameters, tempo (i.e., the pace of a musical piece; McAuley, 2010) has been identified as a key determinant of arousal in adult populations. Music with a faster tempo is typically perceived as more stimulating and increases physiological arousal, whereas slower tempi are perceived as less stimulating (Dillman Carpentier & Potter, 2007; Hofbauer & Rodriguez, 2023; Husain et al., 2002; Ramos et al., 2011). Studies in adults have also demonstrated that tempo interacts with emotional valence. Faster tempi are generally associated with higher perceived emotional valence (i.e., more pleasant), while slower tempi are often linked to a less pleasant evaluation of the music (Bramley et al., 2016; Fernández-Sotos et al., 2016; Husain et al., 2002; Liu et al., 2018; Mote, 2011; Peretz et al., 1998a; Ramos et al., 2011).

Familiarity has been consistently identified as another strong predictor of arousal and emotional valence in adults (Pereira et al., 2011; Van Den Bosch et al., 2013) and, to a lesser extent, in youth samples (Webster & Weir, 2005). Across these age groups, familiar music elicits greater arousal and pleasure than unfamiliar music. This pattern is thought to arise from mechanisms of predictability and anticipation. Indeed, with repeated exposure to musical pieces, listeners learn the structural features of a piece and form expectations about its unfolding (Koelsch, 2012; Pearce & Wiggins, 2012; Tillmann et al., 2000). When these expectations are confirmed, particularly during anticipated and rewarding passages (e.g., a favorite moment of a song), dopaminergic reward circuits are engaged, amplifying both arousal and emotional valence (Ferreri et al., 2019; Salimpoor et al., 2011; Zatorre & Salimpoor, 2013). In adults, familiar and anticipated music engages reward-related brain regions such as the nucleus accumbens and orbitofrontal cortex (Pereira et al., 2011; Salimpoor et al., 2011). Although this neural mechanism has not yet been directly confirmed in adolescents, preadolescent fMRI and behavioral findings suggest that similar reward mechanisms may already be functional during this developmental stage (Fasano et al., 2023; Grewe et al., 2007; Galván, 2010).

Although familiarity generally enhances musical pleasure and arousal, its influence is not unidirectional. Familiarity appears to act as an emotional amplifier, intensifying listeners’ affective responses regardless of their perceived emotional valence, making pleasant excerpts feel more pleasant, but also unpleasant ones more unpleasant (Witvliet & Vrana, 2007). This suggests that familiarity strengthens the emotional impact of music on individuals rather than determining its pleasantness. At the same time, studies have shown that unfamiliar music can also evoke powerful emotional reactions (Fuentes-Sánchez et al., 2022), indicating that emotional engagement does not depend solely on prior exposure. Therefore, these findings suggest that familiarity primarily amplifies the intensity of emotional responses rather than the direction of emotional valence, and that its effects likely vary across listeners according to individual traits such as musical affinity (Eerola et al., 2012; Taruffi et al., 2017; Yik et al., 2023).

### 1.3. Musical Affinity Inter-Individual Variability

In adolescence, music plays a key role in emotional expression, regulation, and identity formation (Biasutti & Habe, 2023; Chong et al., 2024; Miranda et al., 2013; Saarikallio et al., 2014). Although musical training is often used as an index of musical experience and affinity to explain inter-individual variability, it only captures a narrow part of adolescents’ engagement with music. Recent work has therefore proposed moving beyond formal training, and advocates for a multidimensional view of musical engagement (MacGregor & Müllensiefen, 2019; Müllensiefen et al., 2014; Zentner et al., 2025). These frameworks highlight that musical engagement extends beyond instrumental practice to encompass diverse components, such as perceptual sensitivity and emotional responsiveness. In line with this perspective, the present study operationalizes musical affinity through three complementary components of this engagement: (1) musical experience, reflecting exposure and practice; (2) listening diversity, indexing the breadth of musical genres adolescents engage with; and (3) receptivity to musical emotions, capturing the affective sensitivity experienced when listening to music.

#### 1.3.1. Musical Experience

Traditionally, musical training has been regarded as the primary determinant of music-related abilities (Macnamara et al., 2014). Such training can enhance perceptual sensitivity to musical structure and emotional expression (Akkermans et al., 2019; Bailey & Penhune, 2013; Castro & Lima, 2014; Penhune, 2011). However, growing evidence suggests that untrained individuals may still display strong emotional sensitivity due to environmental and individual differences (Larrouy-Maestri et al., 2017; Martins et al., 2021; Mosing et al., 2014; Schellenberg & Lima, 2024). Importantly, adolescents generally have fewer years of accumulated training than adults, simply because of their younger age. Yet, music still plays a central role in their lives (Bonneville-Roussy et al., 2013; Saarikallio et al., 2014). Thus, measuring musical experience can provide useful insights but should not be considered the sole source of musical abilities, including perception of emotions in music.

#### 1.3.2. Listening Diversity

Listening to a broad range of musical genres increases the amount of structural and affective cues to which listeners are accustomed. Such diversity has been linked to stronger preferences and more heterogeneous emotional responses (Bonneville-Roussy et al., 2013; Rentfrow et al., 2011). In adolescents, listening habits have also been associated with emotional well-being (Miranda & Gaudreau, 2011) and with coping styles during stress, suggesting that how youth listen (in addition to what they listen to) influences their emotional outcomes (Miranda & Claes, 2009). Recent work suggests that listening to recommendations of less familiar genres can increase listeners’ openness and curiosity, while also helping to deconstruct pre-existing stereotypes and implicit associations (Porcaro et al., 2024). By expanding the range of musical structures and emotions that feel familiar, listening diversity fosters greater emotional sensitivity and flexibility in music perception, shaping both how individuals respond to music and what kinds of music they prefer.

This relationship between listening diversity, familiarity, and musical preferences is particularly relevant during adolescence, a period when music plays a crucial role in identity formation and emotional expression (Lamont & Hargreaves, 2019). At this developmental stage, preferences are strongly oriented toward popular styles such as pop, hip-hop/rap, electronic dance music (EDM), and contemporary R&B (Bonneville-Roussy et al., 2013; Lamont & Hargreaves, 2019; Lorenzo-Quiles et al., 2020; Magnan, 2016). Therefore, the diversity and distinctiveness of preferred and familiar genres, particularly when preferences extend beyond those typically shared among adolescents, may provide a meaningful indicator of individual differences in musical engagement (Bonneville-Roussy et al., 2013; Martins et al., 2021; Rentfrow et al., 2011).

#### 1.3.3. Receptivity to Musical Emotions

Individuals differ in their sensitivity to the affective content of music. Some are more prone to emotional absorption, defined as a disposition to become deeply immersed in music and to experience strong affective reactions such as chills, tears or intense pleasure (Kreutz et al., 2008; Schäfer et al., 2013). Receptivity reflects a combination of dispositional traits (e.g., openness to experience) and situational engagement (e.g., attentive or background listening), which influence how listeners experience music (Garrido & Schubert, 2011; Juslin & Västfjäll, 2008). Greater receptivity has, in turn, been linked to stronger perceived and felt emotions as individuals with higher openness to experience or greater attentiveness to the music tend to report more intense emotional experiences (Juslin & Västfjäll, 2008; Kreutz et al., 2008; Schäfer et al., 2013).

Together, these three components capture complementary aspects of individual engagement with music and how one responds to it, referred to as musical affinity. Musical affinity encapsulates an individual’s inclination to engage with music through training (e.g., music classes, self-taught), diversity of listening (e.g., listening to multiple musical genres), and receptivity to musical emotion (e.g., propensity to experience emotions during music listening). This conceptualization is especially relevant to adolescence, when everyday musical engagement is both pervasive and emotionally salient (Bonneville-Roussy et al., 2013; Saarikallio et al., 2014).

### 1.4. ADHD and Perception of Musical Emotions

Beyond musical affinity, ADHD constitutes an important individual characteristic that may modulate emotional responses to music (Martin-Moratinos et al., 2023; Saville et al., 2025). ADHD is characterized by inattention, hyperactivity, and impulsivity (American Psychiatric Association, 2022) and often presents with multiple comorbidities, such as psychological or other neurodevelopmental disorders (Biederman et al., 1992; Biederman et al., 2006; Ipçi et al., 2020; Wåhlstedt et al., 2009). Importantly, it is also frequently accompanied by emotion dysregulation, defined as a reduced capacity to effectively modulate emotional states (e.g., rapid mood shifts; Bunford et al., 2015; Christiansen et al., 2019; Graziano & Garcia, 2016). Several authors have argued that these emotional difficulties should occupy a more central place in the conceptualization and diagnosis of ADHD (Barkley, 2010; Hirsch et al., 2018; Musser & Nigg, 2019; Shaw et al., 2014). Given that access to psychological care for youth with ADHD is limited worldwide, identifying accessible strategies for emotional support is crucial (Baweja et al., 2021; Mckenna et al., 2024; Williams et al., 2024; Wright et al., 2015). In this context, engaging with music represents a promising and accessible avenue for supporting the emotional well-being of adolescents with ADHD. Indeed, adolescents with ADHD often exhibit heightened emotional reactivity and may also find in music a resource for emotional and attentional regulation (Graziano & Garcia, 2016; Lachance et al., 2025; Shaw et al., 2014). However, a better understanding of how ADHD influences the perception of musical emotions is essential before music-based intervention can be effectively used in this population. Difficulties in emotional regulation can influence listener’s experience and their perception of musical emotions (Scherer & Zentner, 2001). Recent research suggests that individuals with poorer emotion-regulation abilities engage with music differently and experience altered emotional responses (Kahn et al., 2025; Tan et al., 2024). Moreover, music-based interventions targeting emotional regulation demonstrate measurable improvements in emotion-regulation skills and affective well-being (Vidas et al., 2025). In parallel, evidence indicates that individuals with ADHD often present timing and rhythm-related impairments, such as difficulties synchronizing to a beat or maintaining a steady tempo (Carrer, 2015; Ghanizadeh, 2011; Puyjarinet et al., 2017), which could affect their sensitivity to temporal or arousal-related cues in music, even though improvisational and expressive abilities may remain intact (Groß et al., 2022). Thus, ADHD presents a unique dual profile of emotional dysregulation and atypical temporal processing, making this population particularly relevant for investigating how musical emotions, specifically perceived arousal and emotional valence, are processed and experienced.

Although few studies have examined musical emotion perception, specifically in adolescents with ADHD, related findings provide useful insights. Recent systematic reviews converge in showing that music can play multiple roles in ADHD, spanning music-based therapy, music listening, and performance contexts (Martin-Moratinos et al., 2023; Saville et al., 2025). Furthermore, both active and passive engagement with music appear beneficial for emotional functioning. Active participation (e.g., singing, instrument playing) facilitates emotional expression and self-regulation by engaging rhythmic and motor systems linked to executive control and dopaminergic reward networks (Park et al., 2023; Rickson & Watkins, 2003; Saville et al., 2025). These activities help externalize affective states, promote social connectedness, and reduce aggression or impulsivity. In contrast, passive listening, particularly to calming or familiar music, can modulate arousal and mood by providing structure and predictability, decreasing physiological stress markers (e.g., heart rate variability, cortisol levels), and promoting relaxation (Madjar et al., 2020; Saville et al., 2025; Zimmermann et al., 2019). These findings support the dual role of music as both an expressive and regulatory tool for individuals with ADHD.

Moreover, music-based interventions have shown promising results in reducing core ADHD symptoms, such as hyperactivity and impulsivity, as well as emotion dysregulation by reinforcing attentional focus, promoting rhythmic training, and potentially engaging dopaminergic reward pathways involved in motivation and affective control (Rickson, 2006; Zemestani et al., 2023). However, methodological heterogeneity across studies (e.g., small sample sizes, varied intervention protocols, inconsistent outcome measures) currently limits firm conclusions (Romano et al., 2024).

Taken together, these findings suggest that music may serve both compensatory (by supporting attentional and emotional control) and regulatory (by modulating affective states) functions in ADHD, even in the presence of perceptual challenges (i.e., timing and rhythm difficulties). Nevertheless, the existing literature has largely focused on behavioral and performance outcomes, while the emotional perception of music in ADHD remains insufficiently understood and warrants further investigation. In particular, adolescents’ subjective judgments of musical emotion (i.e., how they perceive arousal and emotional valence in music) remain largely unexplored (Saville et al., 2025), despite their relevance for understanding how music might regulate emotions in this clinical population.

### 1.5. Present Study

**Figure 1:**
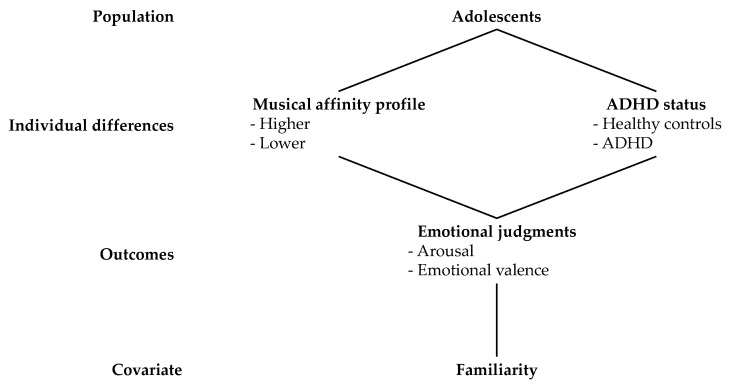
Research design of the present study.

The present online study aims to better understand the perception of musical emotions in adolescents. More specifically, the study investigated how familiarity, musical affinity, and ADHD diagnosis jointly shape adolescents’ judgments of arousal and emotional valence elicited by music. To this end, adolescents rated 55 musical excerpts spanning diverse genres to reflect adolescents’ musical preferences (e.g., pop, hip-hop/rap, EDM) in two dimensions: arousal and emotional valence. Figure 1 presents the research design presented in the study. Building on prior evidence that familiarity strongly predicts emotional responses to music (Pereira et al., 2011; Van Den Bosch et al., 2013), we expected higher familiarity to be associated with greater arousal and with ratings reflecting higher pleasantness on the emotional valence dimension. Based on multidimensional accounts of musical engagement (Müllensiefen et al., 2014; MacGregor & Müllensiefen, 2019), we expected that higher musical affinity, characterized by greater musical experience, listening diversity, and receptivity to musical emotions, would be associated with more polarized emotional ratings. Finally, given the documented impairments of adolescents with ADHD (i.e., timing, rhythm perception, emotion dysregulation), we hypothesized that they would differ from controls in their emotional judgments of arousal and emotional valence, although the literature does not allow for predictions regarding the direction of the effects.

## 2. Materials and Methods

### 2.1. Participants

**Figure 2:**
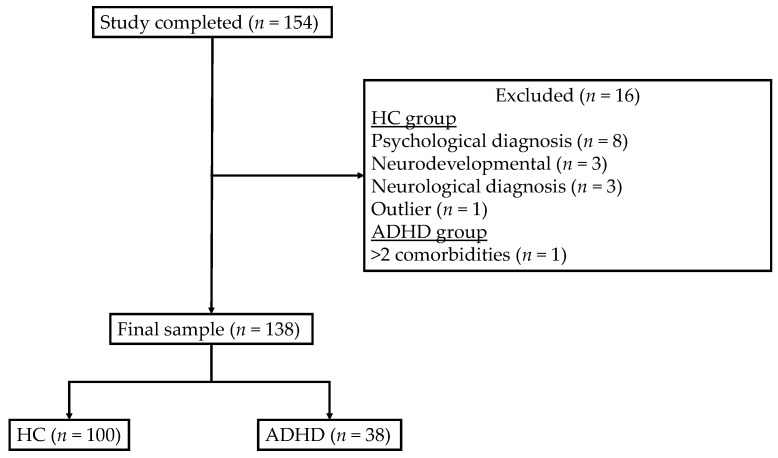
Flowchart of Sample Selection.

Adolescent (12–18 years old) participants were recruited through social media and recruitment posters distributed in schools and nonprofit organizations in Quebec, Canada. To be included in the study, the participants were required to have French as their first or second language and report no auditory or neurological disorder (see Figure 2). Participants were separated into two groups based on their self-reported ADHD diagnosis received from a qualified professional (e.g., physician, psychologist, neuropsychologist): ADHD and healthy controls (HCs). In the ADHD group, a maximum of two psychological or neurodevelopmental diagnoses (e.g., generalized anxiety disorder, learning disorder) was allowed. This exclusion criterion was established to minimize variability in the data while allowing for generalization of the findings as ADHD frequently presents with multiple comorbidities (Biederman et al., 1992; Biederman et al., 2006; Ipçi et al., 2020; Wåhlstedt et al., 2009). For HC participants, exclusion criteria included the presence of psychological and neurodevelopmental disorders. The final sample included 138 adolescents aged 12 to 18 years (M = 14.34, SD = 1.45). Participants had an average of 9 years of education (*SD* = 1.18). Overall, 53.6% identified as girls, 39.9% as boys, and 6.5% as another gender identity. Because the latter proportion was too small for meaningful comparisons, these participants were excluded from analyses involving gender. Most participants reported being North American (76.6%), and the most predominant first language was French (84.8%). Among the 38 ADHD participants, 15 (39.9%) reported comorbid anxiety and/or depression, and 6 (15.8%) had a co-occurring learning disorder.

All adolescents, and their parents for participants under 14 years old, gave their consent to enroll in the study in accordance with the Quebec Civil Code for the participation of minors aged less than 14 years old to minimize risk research. The study was approved by the Comité d’Éthique à la recherche en Éducation et en Psychologie of the University of Montreal.

### 2.2. Materials

#### 2.2.1. Sociodemographic and Health Information

Sociodemographic characteristics, such as age, gender (boy, girl, or other), first and second language, ethnic group, and education were collected in the online form. Health information, such as prior diagnoses of auditory, psychological, neurodevelopmental and neurological problems, was also collected in the online form to verify if they meet the inclusion and exclusion criteria.

#### 2.2.2. Stimuli

Fifty-five instrumental musical pieces were selected from different popular music streaming platforms (e.g., Spotify, iTunes, Youtube; see Appendix A for a complete description of musical pieces). Musical pieces were selected to ensure a variety of musical genres adapted to the preferences of adolescents (e.g., electronic dance music, hip-hop, pop; Bonneville-Roussy et al., 2013; Lamont & Hargreaves, 2019; Lorenzo-Quiles et al., 2020; Magnan, 2016). For each musical piece, an excerpt of 15 seconds was selected. All musical excerpts were normalized at peak value, and logarithmic fade-ins and fade-outs of 500 ms were made using Reaper software v6.78 (Frankel, 2006). All the selected excerpts were composed in a major mode and ranged from 40 to 150 BPM (beats per minute, BPM; *M* = 96.55, *SD* = 27.81). The tempo of each musical piece was first estimated by a trained musician through auditory analysis and subsequently cross-validated with automated values provided by Tunebat (https://tunebat.com/), a Spotify API-based database that provides key and BPM metadata for commercially available recordings. This two-step procedure ensured the reliability of tempo values, particularly for excerpts with less regular rhythmic structures (e.g., solo piano, ambient, orchestral pieces).

#### 2.2.3. Arousal, Emotional Valence, and Familiarity Scales

Participants were asked to rate how they perceived each musical excerpt for arousal, emotional valence, and familiarity using 10-point Likert scales (from 1 to 10; 1 = Relaxing, 10 = Stimulating; 1 = Unpleasant, 10 = Pleasant; and 1 = Unfamiliar, 10 = Familiar).

#### 2.2.4. Musical Affinity

Musical affinity was assessed using three subscales: (A) Musical experience (e.g., years of formal and informal training), (B) Music listening diversity, and (C) Receptivity to musical emotions (e.g., arousal and mood regulation). The internal consistency and descriptive statistics of the subscales are presented in Table 1. See also Appendix B for detailed psychometric properties.

A. Musical Experience

The musical experience subscale was assessed using years of formal and informal musical training as well as the number of instruments played in those two contexts. Response formats were adapted from the Goldsmith Musical Sophistication Index (Gold-MSI; Müllensiefen et al., 2014) to better reflect adolescent experiences (e.g., highest value diminishing from 10+ years to 5+ years). Four items were included: (1) years of formal training, (2) years of informal training (e.g., self-taught, video-based), (3) the number of instruments (including singing) played formally, and (4) the number of instruments played informally. Training duration was rated on a 6-point Likert scale (from 0 = 0 or less than 1 year, to 5 = 5 or more years) and the instrument count used a similar scale (0 = none, 5 = 5 or more).

A composite score was computed by averaging the four items, with higher scores indicating greater and more diverse musical experiences. The subscale demonstrated acceptable internal consistency (Cronbach’s α = 0.803), and corrected correlations from each item with the composite score ranged from 0.719 to 0.792 (*p* < 0.001), suggesting a balanced contribution across indicators. All inter-item correlations were significant (*r* = 0.243 to 0.770, *p* < 0.010), further supporting internal coherence (complete statistical indexes can be found in Appendix B).

B. Music Listening Diversity

Participants reported how frequently they listened to different musical genres (e.g., “How often do you listen to Pop?”) on a 5-point Likert scale (from 1 = Never to 5 = Very often). To obtain a general index of music listening diversity, a two-step procedure was elaborated. First, to isolate musical diversity (i.e., number of genres) from listening frequency (how often the genre is listened to), responses were dichotomized: genres rated as 2 (Rarely) or lower were coded as 0 (Not listened to) and genres rated as 3 (Occasionally) or higher were coded as 1 (Listened to). The resulting music listening diversity index, ranging from 0 to 21, reflected the number of genres that the participants reported having any meaningful engagement with.

Using a coefficient suited for dichotomous items (Kuder-Richardson Formula-20), the index demonstrated good internal consistency (KR-20 = 0.801). Corrected item-total correlations ranged from 0.114 to 0.570. As expected in a formative measure, some genres showed lower correlations due to high prevalence (e.g., pop = 83.3%, hip hop/rap = 81.9%) or rarity (e.g., spiritual/religious music = 12.3%; traditional Quebec music = 27.5%; classical = 29.7%). These items were retained to preserve the conceptual scope of the index, as they reflect unique and meaningful aspects of musical taste, despite their lower statistical contribution.

Second, to create five ordinal levels of musical diversity, participants were ranked based on quintiles derived from the observed distribution of the raw diversity index (range: 0–21). This data-driven approach ensured balanced group sizes, given the asymmetric distribution of the data. The resulting categories ranged from 1 (very low diversity) to 5 (very high diversity).

C. Receptivity to Musical Emotions

This subscale was inspired by a validated questionnaire by Schäfer et al. (2013) to measure participants’ receptivity to musical emotions. Initially composed of 10 items (see Appendix B), one item (“Music makes me feel nostalgic”) was excluded from analysis due to its low item-total Pearson correlation (*r* = 0.297) and weak convergence with the remaining items (*r* = 0.107 to 0.333). Participants rated each item on a 5-point Likert scale (1 = Never, 5 = Very often), and a mean score across the remaining nine items (e.g., “Music comforts me”) was computed. Higher scores indicate greater emotional receptivity to music. The subscale demonstrated good internal consistency (Cronbach’s α = 0.868) and all retained items showed moderate to strong correlations with the total score (ranging from 0.587 to 0.777), supporting construct validity.

D. Total Musical Affinity Score

A total score was computed by summing the values of the three subscales. Receptivity to musical emotions and music listening diversity were both scaled from 1 to 5, while musical experience ranged from 0 to 5. Although the subscales were moderately to strongly intercorrelated (*r* = 0.48 to 0.84), internal consistency for the total score was limited (Cronbach’s α = 0.502), reflecting the conceptual heterogeneity of the subscales. Given these psychometric considerations, the total musical affinity score was retained for descriptive purposes only, rather than for classifying participants by musical affinity.

All subscale distributions showed acceptable levels of skewness and kurtosis (see Table 1), supporting their use as input variables in a cluster analysis to determine meaningful profiles of musical affinity.

### 2.3. Procedure

The online study was constructed using LimeSurvey v3.28.52 (LimeSurvey GmbH, n.d., Hamburg, Germany) and the International Laboratory for Brain, Music, and Sound Research Online Testing Platform (BRAMS-OTP, https://brams.org/category/online-testing-platform/, accessed on 10 November 2025), and was administered in French. After giving their consent to enroll in the study, participants first completed the general information form and musical affinity questionnaire. Then, they rated 55 musical excerpts on three dimensions (arousal, emotional valence, familiarity). Before the beginning of this part, participants were asked to wear headphones to reduce environmental distractions, to complete the study in a calm environment, and to adjust the volume intensity to a comfortable level. To ensure participants’ comprehension of the task requirements, two practice trials were administered. Participants could replay each musical excerpt as many times as needed. Excerpts were presented in a randomized order for each participant.

### 2.4. Data Analysis

#### 2.4.1. Musical Affinity Classification

To identify participant profiles based on musical affinity, the three subscales (musical experience, music listening diversity, and receptivity to musical emotions) were entered into a two-step cluster analysis. This approach was selected to empirically distinguish participants with lower and higher musical affinity while accounting for the multidimensional nature of the construct.

While the automatic procedure of the analysis proposed three clusters (silhouette measure ≈ 0.40), a two-cluster solution was retained, as it provided the highest quality (fair-to-good; silhouette measure ≈ 0.50) and offered clearer interpretability. To verify the stability of the classification, a k-means structure was also computed, with strong convergence (χ^2^(1) = 96.11, *p* < 0.001) and an excellent agreement (κ = 0.82, *p* < 0.001) between methods. The resulting profiles were labeled Higher Musical Affinity (HMA) and Lower Musical Affinity (LMA).

#### 2.4.2. Preliminary Analyses

Descriptive statistics and two-way ANOVAs (Musical affinity profile × Diagnostic group) were conducted to compare subgroups on demographic variables (gender, age, years of education) and musical affinity scores (each subscale and total composite score). These analyses served to verify group equivalence and to ensure that the musical affinity classification was independent of diagnostic status.

#### 2.4.3. Main Analyses

Two separate univariate analyses of covariance (ANCOVAs) were conducted to examine the effects of musical affinity profile (lower vs. higher) and diagnostic group (ADHD vs. HC) on standardized ratings of arousal and emotional valence (z-scores). In each model, standardized familiarity ratings (z-scores) were entered as a covariate to control for the well-established influence of familiarity on emotional responses to music. The assumptions of the ANCOVA were tested (Pearson correlations, Levene’s test, homogeneity of regression slopes) prior to analysis to ensure their applicability. Additional linear regressions were conducted when necessary to further investigate significant interaction effects.

All analyses were conducted using SPSS Statistics Version 28.0.1.0 (IBM Corp., Armonk, NY, USA), with a two-tailed significance level of α = 0.05 (or adjusted α for multiple comparisons). Post hoc analyses were conducted when significant effects were observed, with Bonferroni-adjusted p-values reported and interpreted against α = 0.05.

## 3. Results

### 3.1. Contrasting ADHD and Healthy Controls Across Musical Affinity Profiles

#### 3.1.1. Sample Characteristics

To examine the effects of musical affinity profile (LMA vs. HMA) and diagnostic group (ADHD vs. HC), two-way ANOVAs were conducted on sociodemographic variables and musical affinity scales (Table 2). No significant effects or interactions emerged for age or education, indicating that the four subgroups were comparable for these characteristics. However, the gender distribution differed across subgroups (χ^2^(3) = 17.21, *p* < 0.001, *V* = 0.37), with girls being overrepresented in the ADHD-HMA group and underrepresented in the HC-LMA group.

#### 3.1.2. Musical Affinity Profiles

As expected, the HMA profile reported significantly higher scores on all three musical affinity subscales and on the composite score compared to the LMA profile (all *p*s < 0.001; see Table 2). No significant main effects of diagnosis or interaction effects with diagnostic group were observed on any musical affinity measure, suggesting that ADHD status did not influence musical experience, listening diversity, or receptivity to musical emotions.

In summary, the two-step clustering yielded two robust and interpretable musical affinity profiles that were balanced across diagnostic groups and comparable in age and education, though the gender distribution varied. These profiles served as the basis for the subsequent analyses on emotional judgments (arousal and emotional valence).

### 3.2. Impact of Individual Differences on Arousal and Emotional Valence

#### 3.2.1. Preliminary Associations Between Arousal, Emotional Valence, Familiarity, and Individual Differences

Given the unequal gender distribution across diagnostic groups, musical affinity profiles, and their crossed subgroups, preliminary analyses were conducted to assess its potential influence on the main analyses. Specifically, a three-factor ANCOVA was performed with diagnostic group, musical affinity profile, and gender as between-subject factors. This analysis aimed to verify whether gender acted as a confounding variable. No interactions involving gender were observed (all *p*s > 0.05, all η^2^_p_ < 0.012). Consequently, gender was not retained as a factor in the main analyses.

Pearson correlations indicated that familiarity was positively associated with evaluations of musical excerpts based on arousal (*r* = 0.27, *p* = 0.001), emotional valence (*r* = 0.49, *p* < 0.001), and musical affinity profile (*r* = 0.30, *p* < 0.001), supporting its inclusion as a covariate. No significant association was observed with diagnostic group (*p* = 0.652).

#### 3.2.2. Arousal

**Figure 3:**
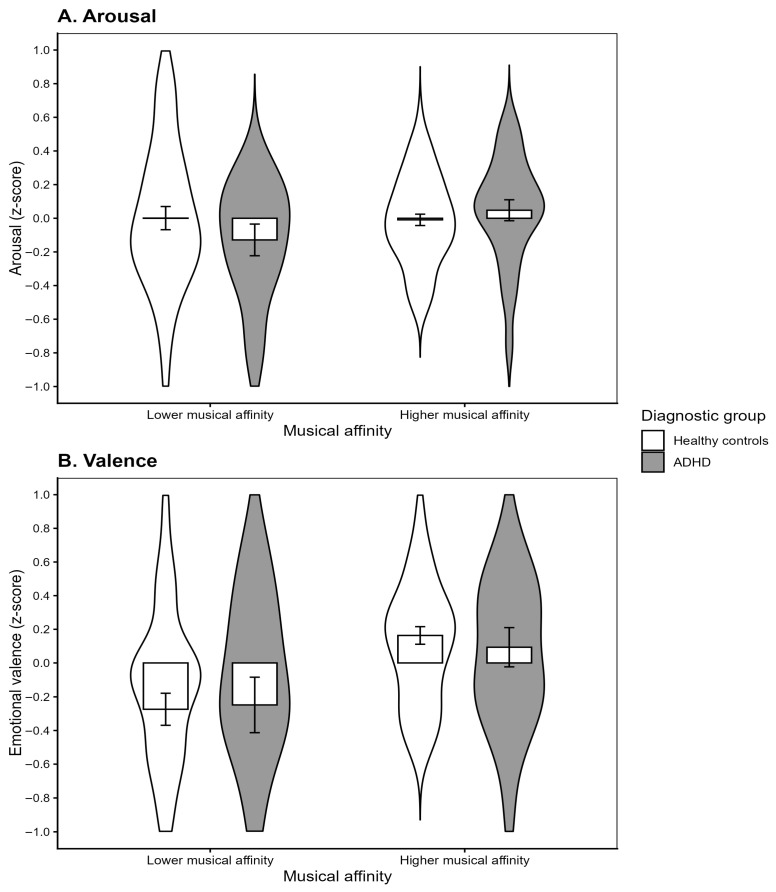
Mean and standard errors for (**A**) arousal and (**B**) emotional valence ratings as a function of musical affinity profiles and ADHD diagnosis.

A two-way ANCOVA with diagnostic group and musical affinity profile as between-subject factors, and familiarity entered as a covariate (see Table 3), revealed a significant main effect of familiarity on arousal ratings (*F*(1, 133) = 9.96, *p* = 0.002, η^2^_p_ = 0.070), indicating that more familiar excerpts were generally perceived as more arousing. No main effects of diagnostic group (*p* = 0.721) or musical affinity (*p* = 0.764) were found, and their interaction did not reach significance (*p* > 0.05; see Figure 3A). Thus, arousal ratings were primarily driven by familiarity, with no evidence for moderating effects of diagnostic group or musical affinity.

#### 3.2.3. Emotional Valence

The homogeneity of regression slopes assumption was not fully met for emotional valence (see Table 3), as familiarity interacted significantly with musical affinity profile (*F*(1, 131) = 5.64, *p* = 0.019, η^2^_p_ = 0.041) and marginally with diagnostic group (*F*(1, 131) = 3.20, *p* = 0.076, η^2^_p_ = 0.024). Therefore, these interaction terms were retained in the main analysis to ensure compliance with ANCOVA assumptions and because they involved independent variables central to the study. Each interaction was explored with regression analyses.

The ANCOVA revealed a strong main effect of familiarity (*F*(1, 131) = 34.46, *p* < 0.001, η^2^_p_ = 0.208), indicating that more familiar excerpts were perceived as more pleasant. In contrast, the main effects of musical affinity profile (*F*(1, 131) = 1.70, *p* = 0.195, η^2^_p_ = 0.013) and diagnostic group (*F*(1, 131) = 0.46, *p* = 0.498, η^2^_p_ = 0.004), as well as their interaction (*F*(1, 131) = 3.00, *p* = 0.085, η^2^_p_ = 0.022), were not significant.

**Figure 4:**
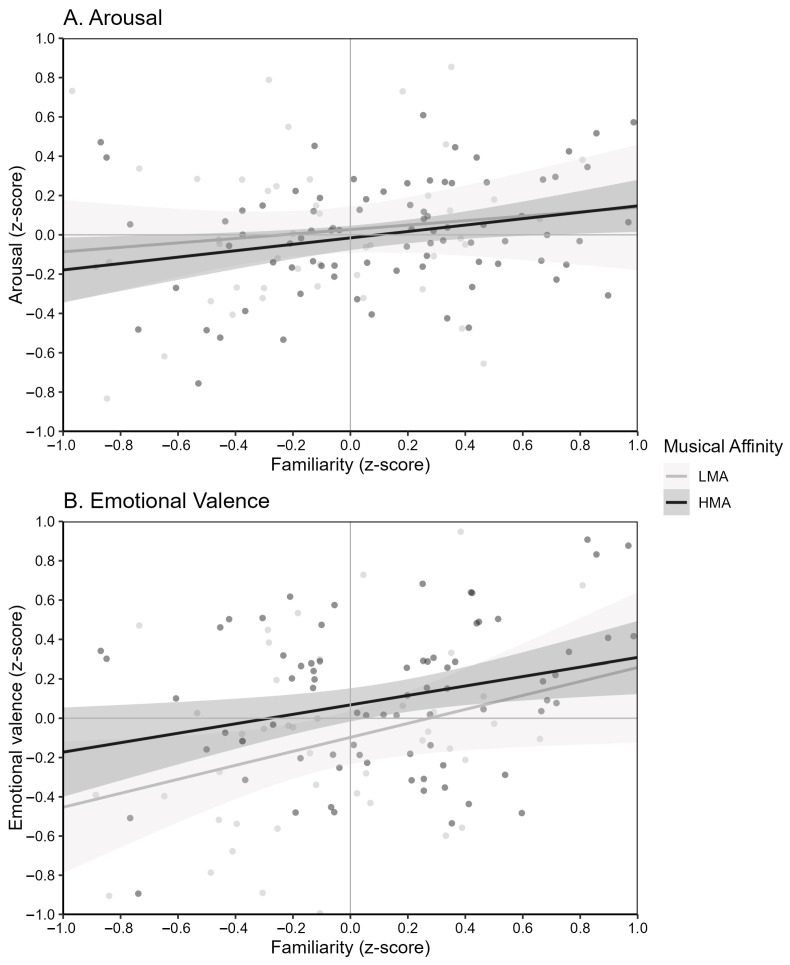
Effects of familiarity on (**A**) arousal and (**B**) emotional valence ratings by musical affinity profile.

Follow-up regression analyses (see Figure 4) confirmed the significant interaction between familiarity and musical affinity profile (β = −0.27, *p* = 0.028), suggesting that the positive association between familiarity and emotional valence was more pronounced among participants in the LMA group (β = 0.57, *p* < 0.001) compared to those in the HMA group (β ≈ 0.30, *p* > 0.05). In contrast, the interaction between diagnostic group and familiarity was only marginal in the ANCOVA and was not supported by the regression (β = 0.10, *p* = 0.242).

## 4. Discussion

The present study aimed to investigate how diagnostic status (ADHD vs. controls), musical affinity (LMA vs. HMA), and familiarity with musical excerpts shape adolescents’ perception of musical emotions. Participants completed tests of musical affinity (musical experience, music listening diversity, and receptivity to musical emotions) and rated 55 musical excerpts in terms of perceived arousal, emotional valence, and familiarity. The data of 138 adolescents was analyzed, including 38 ADHD and 100 healthy controls. Based on a cluster analysis including the three musical affinity subscales, 50 participants were classified as having lower musical affinity (LMA) and 88 as having higher musical affinity (HMA), resulting in four diagnostic x affinity subgroups: ADHD-LMA, ADHD-HMA, HC-LMA, and HC-HMA. Overall, the results identified familiarity as the strongest predictor of adolescents’ emotional judgments to music. Furthermore, musical affinity influenced the effect of familiarity on emotional valence judgments, with adolescents in the LMA profile showing a stronger association than those in the HMA profile. However, diagnostic status (ADHD vs. HC) had no significant effect on participants’ emotional judgments.

### 4.1. Familiarity at the Core of Musical Emotion

Familiarity emerged as the most robust predictor of both arousal and emotional valence, with stronger effects on emotional valence in the LMA profile. This result is partly consistent with our expectations, as prior research has established familiarity as a central determinant of musical liking and emotional response (Pereira et al., 2011; Van Den Bosch et al., 2013). Our findings allowed to refine this association by highlighting variability in how familiarity supports emotional judgments based on individual characteristics.

The results align with the mere exposure effect (Zajonc, 1968), which posits that repeated encounters with a stimulus increase preference, and with Berlyne’s (1971) theory of aesthetic experience, which emphasizes that pleasure arises from a balance between familiarity and novelty (Peretz et al., 1998b). From these perspectives, LMA adolescents may require higher familiarity to experience music as pleasant, reflecting a narrower optimal zone for novelty. By contrast, HMA adolescents, who exhibit greater listening diversity, appear more open to novelty, enabling them to derive positive affect even from less familiar excerpts. One mechanism through which familiarity may exert its effects is autobiographical memory, as familiar music more readily evokes personal memories and associated responses (Jakubowski & Eerola, 2022; Juslin & Västfjäll, 2008). This suggests that adolescents with lower musical affinity have fewer opportunities to form positive autobiographical associations with a wide range of musical genres and may find it harder to appreciate less familiar music.

Two interaction effects approached significance in the ANCOVA on emotional valence: diagnostic group × familiarity (*p* = 0.076) and diagnostic group × musical affinity (*p* = 0.085). Although these trends did not reach statistical significance, they point toward potential group-specific patterns in how music is emotionally processed in ADHD. Given the limited power of the present sample to detect higher-order interactions, these trends should be interpreted cautiously and warrant replication in larger samples.

Finally, arousal ratings were predicted by familiarity but did not vary according to diagnostic status or musical affinity level. Research shows that arousal perception is primarily driven by musical parameters such as tempo (Husain et al., 2002). Familiarity may nonetheless enhance these effects by strengthening prediction and reward processes during listening (Pereira et al., 2011; Van Den Bosch et al., 2013) independently of diagnostic status or musical affinity profile.

### 4.2. Rethinking Emotional Responses to Music in ADHD

No significant differences were found between ADHD and HC participants in their ratings of perceived arousal, emotional valence, or familiarity, indicating that adolescents with ADHD perceived musical emotions comparably to controls. This finding contradicts our initial hypothesis that ADHD participants would differ from controls in their emotional judgement.

It is useful to situate this result within existing integrative models conceiving musical emotion processing as a multistage mechanism (Brattico & Pearce, 2013; Juslin & Västfjäll, 2008). The first stage, early acoustic-musical processing, involves the perceptual decoding of musical parameters (e.g., tempo, mode, rhythm, timbre), which provide the foundation for subsequent emotional appraisal. The second stage, perceived emotion, refers to the cognitive evaluation of what the music expresses (e.g., pleasant vs. unpleasant, stimulating vs. relaxing) without necessarily eliciting the corresponding emotion in the listener (Eerola & Vuoskoski, 2013; Schubert, 2013). The third stage, felt emotion, concerns the listener’s own emotional experience induced by the music, encompassing physiological arousal, chills, pleasure, or mood changes (Juslin & Västfjäll, 2008). Finally, a fourth stage, sensorimotor engagement, involves behavioral synchronization and motor reproduction, such as rhythm reproduction, synchronization, or active engagement, which are known to be affected in ADHD (Groß et al., 2022; Hove et al., 2017; Puyjarinet et al., 2017). Considering this multistage mechanism, the absence of diagnostic differences in the present study suggests that difficulties commonly reported in emotional regulation and temporal processing among individuals with ADHD (Ghanizadeh, 2011; Graziano & Garcia, 2016; Shaw et al., 2014) were not reflected under the current experimental conditions. This may be because our paradigm focused specifically on stage 2, that is, the cognitive appraisal of perceived emotion, rather than on the induction, regulation, or motor synchronization processes typically associated with later stages of musical emotion processing. This pattern may indicate that, within our sample and the musical pieces selected, the cognitive processes involved in recognizing emotions in music rely on relatively automatic perceptual mechanisms that remain preserved in ADHD, even when difficulties arise in later stages of emotional regulation or motor synchronization.

### 4.3. Musical Affinity: A Gateway to Adolescents’ Music Experience

Adolescents with higher musical affinity rated the excerpts as more pleasant and more familiar than their lower-affinity peers, while arousal ratings did not differ between profiles. This pattern partially supports our hypothesis, indicating that musical affinity primarily influenced emotional valence rather than perceived arousal.

One plausible explanation for this pattern lies in the role of musical absorption, defined as the tendency to become deeply immersed in and emotionally affected by music (Kreutz et al., 2008). Adolescents with higher musical affinity likely experience stronger absorption (Kreutz et al., 2008; Schäfer et al., 2013), enabling them to perceive positive affect even with less familiar excerpts. In contrast, lower-affinity adolescents may rely more strongly on familiarity to trigger comparable levels of emotional appreciation (Lahdelma & Eerola, 2020; Pereira et al., 2011; Van Den Bosch et al., 2013).

Neurophysiological evidence on music reward provides converging support that familiarity and emotional engagement jointly enhance pleasure during music listening. Familiar and emotionally engaging music have been shown to activate dopaminergic reward circuits in the striatum and medial prefrontal cortex (Brattico et al., 2025; Pereira et al., 2011), and meta-analytic evidence confirms that familiarity modulates neural responses in these regions (Freitas et al., 2018). Moreover, dopamine release is strongest when listening to familiar and emotionally engaging music (Salimpoor et al., 2011; Salimpoor & Zatorre, 2013). Although the present study did not assess neural activity, the existing evidence suggests that familiarity and emotional engagement jointly recruit reward-related mechanisms. It is therefore plausible that adolescents with higher musical affinity may more readily recruit these systems even when listening to unfamiliar music, possibly due to their greater openness to experience, emotional absorption and diversity of their musical habits. In contrast, adolescents with lower musical affinity may depend more on familiarity to engage these same mechanisms and experience similar levels of pleasure.

### 4.4. Harnessing Familiarity in Educational, Clinical, and Self-Care Contexts

These findings carry practical implications for both musical education and therapeutic contexts. The stronger reliance of LMA adolescents on familiarity suggests that limited exposure to musical diversity constrains the range of positive emotional judgments they derive from music. In contrast, HMA adolescents, who report more varied listening habits, appear better disposed to appreciate and emotionally engage with even unfamiliar excerpts.

In educational (e.g., school) or clinical settings (e.g., music-based interventions), fostering diverse listening experiences could therefore enhance adolescents’ emotional engagement and flexibility in music-based activities (Saarikallio et al., 2014). Exposure to a variety of genres and cultural traditions may expand the emotional spectrum accessible through music while supporting curiosity, openness, and the development of more adaptive regulation strategies (Demorest et al., 2008; Morrison & Demorest, 2009). Such exposure may not only enrich musical appreciation but also broaden the emotional benefits of music, supporting adolescents’ capacity to use music as a flexible tool for regulation.

Beyond structured interventions, these insights also highlight the potential of familiarity as a self-regulatory resource. Encouraging adolescents, particularly those with lower musical affinity, to intentionally use familiar and emotionally positive music in everyday life may strengthen their ability to modulate mood, reduce stress, and engage in self-care through music. Over time, combining familiar selections with gradual exploration of new styles could balance comfort and novelty, promoting both emotional stability and personal growth.

### 4.5. Strengths, Limitations and Future Studies

A core strength of the present study lies in its integration of diagnostic status, musical affinity, and familiarity within the same design, allowing for a comprehensive examination of individual differences in adolescents’ emotional responses to music. Another important strength of the study resides in the control of potential confounding variables, which increases confidence in the validity of the observed effects. In addition, the choice of musical excerpts was tailored to the musical preferences of the adolescent population, including genres such as pop, hip-hop/rap, and EDM, rather than relying on classical music excerpts that are more commonly used in musical emotion research (Eerola & Vuoskoski, 2013). This enhances both the ecological validity and the relevance of the findings for the age group in this study. Finally, although the validation of our musical affinity questionnaire was not the focus of the present analyses, it represents an attempt to capture adolescents’ overall engagement with music across three components adapted to their reality. While this composite measure demonstrated acceptable reliability, future work is needed to validate and refine the construct, particularly to determine its specificity and generalizability beyond the present sample.

Several limitations must be noted. First, emotional judgments were measured exclusively through self-report, which may not fully capture the complexity of affective experience. Future work should strengthen this approach by combining self-reports with physiological measures, such as electrodermal conductance or heart rate variability, which provide complementary indices of physiological arousal. Second, the sample was drawn from a relatively homogeneous cultural context, which limits the generalizability of the findings, as cultural familiarity is known to strongly influence emotional responses to music. Third, the sample was unbalanced in terms of gender, with a higher proportion of girls overall and across certain subgroups, which limits the interpretation of gender effects.

Other musical parameters, such as mode, timbre, and harmonic complexity, also contribute to emotional appraisal and should be considered alongside tempo (Gabrielsson & Lindström, 2010; Vieillard et al., 2008). Additional measures of rhythm and timing performance would also be valuable (e.g., BAASTA; Dalla Bella et al., 2017, 2024) given their relevance to arousal and music perception and their known alteration in ADHD. Expanding recruitment to more culturally diverse populations would be useful to clarify the role of cultural familiarity. In addition, efforts should be made to ensure more representative gender distributions to explore whether gender moderates the relationship between diagnosis, musical affinity, and emotional responses to music (Hartung et al., 2025). Including participants’ preferred music alongside standardized excerpts may further enhance ecological validity and provide insight into how familiarity and musical affinity operate in real-world contexts.

## 5. Conclusions

This study provides novel evidence on how adolescents’ perception of musical emotions are affected by familiarity, musical affinity, and diagnostic status. While ADHD and healthy control participants did not differ in their ratings of arousal or emotional valence, familiarity emerged as a robust predictor across groups, particularly among adolescents with lower musical affinity. These findings suggest that the emotional impact of music in adolescence is shaped less by diagnostic status than by the interplay between familiarity and individual engagement with music. By adopting a multidimensional measure of musical affinity, this study underscores the value of considering diverse forms of musical involvement beyond formal training. Collectively, our results highlight the central role of familiarity in shaping emotional experience during music listening, as well as its potential benefits for music-based interventions and the well-being of adolescents with or without ADHD.

## Figures and Tables

**Table 1:** Descriptive Statistics and Internal Consistency of the Musical Affinity Subscales and Composite Score.

Measure	Number of Items	Scale Range	*M* (*SD*)	Min–Max	Skewness/ Kurtosis	Reliability Coefficient
Musical experience	4	0–5	0.84 (1.04)	0.00–4.75	1.50/2.00	α = 0.742
Music listening diversity	21 (dichotomized)	0–21 (raw)	7.01 (4.04)	1–18 (raw)	−0.17/−1.11	KR-20 = 0.801
		1–5 (ordinal)	3.18 (1.33)	1–5 (ordinal)		
Receptivity to musical emotions	9	1–5	3.82 (0.75)	1.44–5.00	−0.57/0.43	α = 0.868

**Table 2:** Descriptive Statistics and Two-Way ANOVA Results for Demographic and Musical Affinity Variables by Musical Affinity Profile and Diagnostic Group.

	HC-LMA (*n* = 37)	HC-HMA (*n* = 63)	ADHD-LMA (*n* = 13)	ADHD-HMA (*n* = 25)	Main Effect Musical Affinity			Main Effect Diagnosis			Interaction Musical Affinity × Diagnosis		
Variable	*M* (*SD*)	*M (SD)*	*M* (*SD*)	*M* (*SD*)	*F*	*p*	η^2^_p_	*F*	*p*	η^2^_p_	*F*	*p*	η^2^_p_
Sociodemographics													
Gender (% girls)	35.3%	61.0%	41.7%	87.5%	–	–	–	–	–	–	–	–	–
Age (years)	14.00 (1.05)	14.33 (1.54)	14.54 (1.33)	14.76 (1.74)	0.920	0.339	0.007	2.79	0.097	0.020	0.04	0.847	0.000
Education (years)	8.86 (0.95)	8.94 (1.33)	9.15 (0.80)	9.24 (1.23)	0.112	0.738	0.001	1.58	0.211	0.012	0.00	0.978	0.000
Musical affinity scales													
Musical experience (/5)	0.36 (0.45)	1.04 (1.14)	0.17 (0.34)	1.39 (1.21)	23.953	<0.001	0.152	0.18	0.674	0.001	2.00	0.159	0.015
Music listening diversity (/5)	1.81 (0.74)	3.97 (0.86)	1.92 (0.76)	3.88 (1.09)	140.28	<0.001	0.511	0.01	0.945	0.000	0.33	0.565	0.002
Receptivity to musical emotions (/5)	3.23 (0.80)	4.13 (0.52)	3.38 (0.67)	4.16 (0.54)	45.45	<0.001	0.253	0.56	0.454	0.004	0.21	0.652	0.002
Musical affinity total (/15)	5.40 (1.13)	9.13 (1.35)	5.47 (0.65)	9.43 (1.81)	204.62	<0.001	0.604	0.49	0.487	0.004	0.19	0.667	0.001

Note. HC = Healthy Control; ADHD = Attention-Deficit/Hyperactivity Disorder; LMA = Lower Musical Affinity; HMA = Higher Musical Affinity. All *F* tests have 1 and 134 degrees of freedom.

**Table 3:** Univariate ANCOVAs for arousal and emotional valence judgments: Effects of diagnosis and musical affinity group controlling for familiarity.

Dependent Variable	Effect	*F*	*df*1	*df*2	*p*	η^2^_p_
Arousal	Familiarity (cov.)	9.96	1	133	0.002 **	0.070
	Diagnosis	0.13	1	133	0.721	0.001
	Musical affinity	0.09	1	133	0.764	0.001
	Diagnosis × Musical affinity	1.58	1	133	0.212	0.012
Emotional valence	Familiarity (cov.)	34.46	1	131	<0.001 ***	0.208
	Diagnosis	0.46	1	131	0.498	0.004
	Musical affinity	1.70	1	131	0.195	0.013
	Diagnosis × Musical affinity	3.00	1	131	0.085	0.022
	Diagnosis × Familiarity	3.20	1	131	0.076	0.024
	Musical affinity × Familiarity	5.64	1	131	0.019 *	0.041

Note. * *p* < 0.05; ** *p* < 0.01; *** *p* < 0.001. Cov. = Covariate.

## Data Availability

The data presented in this study are available on request from the corresponding author due to privacy reasons.

## Abbreviations

The following abbreviations are used in this manuscript:

ADHD	Attention-Deficit/Hyperactivity Disorder
HC	Healthy Controls
LMA	Lower Musical Affinity
HMA	Higher Musical Affinity

## Appendix A

This appendix lists the 55 musical excerpts used in the study, ordered by tempo (BPM). For each track, we report the Title—Artist (Year), tempo, musical genre (aligned with the 21-genre inventory used in our measures), and the excerpt timing (start–end, mm:ss). Musical genre labels are pragmatic, intended for analytic grouping rather than formal musicological classification.

**Table A1:** Musical excerpts with tempo (BPM) and precise/broad genre classification.

Title—Artist (Year)	Tempo	Musical Genre	Excerpt Timing
Sugarcane—Ana Olgica (2017)	40	Hip-hop/Rap	00:19–00:31
Arrival of the Birds—The Cinematic Orchestra & LMO (2008)	44	Film/Video game soundtracks	00:38–00:52
Her Eyes the Stars—LUCHS, P.B. Almkvisth (2016)	47	Classical	00:19–00:31
Time—Hans Zimmer (2010)	60	Film/Video game soundtracks	00:05–00:16
A Long Way Home—Jack Wall (2013)	65	Film/Video game soundtracks	00:18–00:31
Yes, I Am a Long Way from Home—Mogwai (1997)	65	Alternative	01:09–01:23
Deep Blue Day—Brian Eno (1983)	70	Electronica	00:27–00:41
Eternal Youth—RUDE (2016)	70	Hip-hop/Rap	00:26–00:38
River Flows in You—Yiruma (2001)	70	Classical	00:33–00:47
Front Yard—Trixie Muff (2020)	75	Indie	00:06–00:20
Quilted Hills—Ocha & Laffey (2019)	75	Hip-hop/Rap	00:13–00:26
Ocean—Gradient Islant (2020)	75	Hip-hop/Rap	00:11–00:25
Underground Vibes—DJ Cam (1995)	75	Hip-hop/Rap	00:14–00:28
Daffodils—Guustavv (2018)	76	Hip-hop/Rap	00:11–00:25
Gendèr—Makoto San (2020)	77	Other cultural traditions	00:12–00:25
Nostrand Ave—Tom Doolie (2020)	80	Hip-hop/Rap	00:10–00:24
Spirals—Slowheal (2018)	80	Hip-hop/Rap	00:23–00:37
Saturday Afternoon—Sweet Oscar (2020)	80	Hip-hop/Rap	00:12–00:25
You—Petit Biscuit (2015)	80	EDM	00:11–00:25
Tuesday, July 14—Dogman Chill (2020)	82	Hip-hop/Rap	00:11–00:25
Go to Shanghai—Atrisma (2020)	83	Jazz/Blues	00:08–00:22
Lie Down—Kino B (2020)	85	Hip-hop/Rap	00:22–00:35
Norr—Polaroit (2020)	85	Indie	01:09–01:22
Mixed Feelings—DONTCRY, Glimlip (2019)	86	Hip-hop/Rap	00:12–00:26
Cumbia del Olvido—Nicola Cruz (2015)	86	Latin	00:55–01:04
Phew—Jules Hiero, Antônio Neves, Ed Santana (2020)	87	Jazz/Blues	00:33–00:47
Awake—Tycho (2014)	88	Electronica	00:19–00:33
Bewildered—Hanz (2019)	90	Electronica	00:42–00:55
Smokes—Monkey Larsson (2020)	90	Hip-hop/Rap	00:10–00:24
Plus tôt—Alexandra Stréliski (2018)	90	Classical	00:49–00:53
Golden Crates—Dusty Deck (2020)	90	Hip-hop/Rap	00:03–00:17
Noctuary—Bonobo (2003)	92	Electronica	00:39–00:50
Poetic Wax—Dusty Deck (2020)	97	Hip-hop/Rap	00:09–00:23
Intro—The xx (2009)	100	Indie	00:39–00:53
Invincible—DEAF KEV (2015)	100	EDM	00:10–00:24
Hello—OMG (2014)	107	K-pop	00:00–00:14
Multi—Lstn (2019)	117	Electronica	00:08–00:22
Sumatra—Nora Van Elken (2019)	118	EDM	00:18–00:32
Rest in Peace—Super Duper (2018)	120	EDM	00:12–00:26
Ventura—City of the Sun (2018)	120	Alternative	00:24–00:37
Ether—Mogwai (2016)	120	Alternative	01:10–01:24
The Morning After—Anthony Favier (2019)	122	Jazz/Blues	00:31–00:45
Say Goodbye—Lucas De Mulder & The New Mastersounds (2020)	128	Disco/Funk	00:01–00:15
Bot—Deadmau5 (2009)	130	EDM	00:00–00:14
Candyland—Tobu (2015)	130	EDM	00:54–01:08
Firefly—Jim Yosef (2015)	130	EDM	00:31–00:45
Nekozilla—Different Heaven (2016)	130	EDM	00:13–00:27
Nova—Ahrix (2013)	130	EDM	00:40–00:54
The Last Day—City of the Sun (2020)	130	Alternative	00:59–01:13
Slumber Party—Sonny Side Up (2017)	132	Hip-hop/Rap	00:14–00:28
Silly Boy—Jack Wall (2011)	135	Film/Video game soundtracks	00:28–00:42
Everything—City of the Sun (2016)	136	Alternative	00:52–01:04
I’ll Tell You Someday—Plini (2020)	140	Metal	02:08–02:24
In the House of Tom Bombadil—Nickel Creek (2000)	150	Folk	00:00–00:14
Assassin—The Fearless Flyers (2020)	150	Disco/Funk	00:08–00:22

## Appendix B

Musical affinity was operationalized through three complementary subscales: Musical Experience, Music Listening Diversity, and Receptivity to Musical Emotions. Each subscale was scored on a 0–5 or 1–5 scale to ensure comparability across indices. Detailed items, sources, and adaptations are presented below.

### Appendix B.1. Musical Experience Subscale

The items of the Musical Experience subscale were adapted from the *Goldsmith Musical Sophistication Index* (Gold-MSI; Müllensiefen et al., 2014) to better capture adolescent musical engagement and to harmonize the scoring with the other subscales of the musical affinity index. The adapted version also sought to include formal and informal musical experience. The table below presents the inter-item and item-total corrected correlations.

**Table A2:** Musical Experience: Inter-Item Correlations (Lower Triangle) and Corrected Item–Total Correlations.

Item	Years of Formal Training	Years of Informal Training	Number of Instruments (Formal)	Number of Instruments (Informal)	Item-Total *r* (Corrected)
Years of formal training	—				0.620
Years of informal training	0.349	—			0.607
Number of instruments (formal)	0.870	0.299	—		0.605
Number of instruments (informal)	0.291	0.925	0.298	—	0.639

### Appendix B.2. Music Listening Diversity Subscale

The list of genres was adapted from the *Cultural Practices in Québec Survey* (see table 47 in Magnan, 2016) and expanded to include contemporary categories relevant to adolescents (e.g., K-pop, EDM, film soundtracks). Participants rated each genre on a 5-point scale (1 = Never, 2 = Rarely, 3 = Occasionally, 4 = Often, 5 = Very often). Responses ≥ 3 were dichotomized as “listened to” and summed across 21 genres to create a raw diversity index (range: 0–21). To harmonize with the other subscales and avoid overweighting, the raw score was recoded into five levels according to sample distribution: Very low (0–2), Low (3–4), Moderate (5–7), High (8–10), Very high (11+) diversity. The table below presents the range of inter-item correlations and item-total corrected correlations.

**Table A3:** Music Listening Diversity: Range of Inter-Item Correlations and Corrected Item–Total Correlations.

Item	Inter-Item *r* (min)	Inter-Item *r* (max)	Item–Total *r* (Corrected)
Classical	−0.065	0.272	0.197
Punk	−0.170	0.346	0.416
Electronica	−0.051	0.585	0.324
EDM (Electronic Dance Music)	0.038	0.585	0.413
Pop	−0.060	0.248	0.321
Rock	−0.056	0.538	0.443
Indie	−0.163	0.447	0.389
Metal	−0.211	0.538	0.358
Jazz/Blues	−0.071	0.473	0.322
Alternative	−0.146	0.447	0.474
Hip hop/Rap	−0.211	0.206	0.018
R&B	−0.042	0.282	0.395
Country/Western	−0.060	0.473	0.344
Disco/Funk	−0.013	0.436	0.483
K-pop	−0.060	0.358	0.422
Latin	−0.021	0.403	0.421
Folk	−0.063	0.413	0.455
Film/Video game soundtracks	−0.100	0.314	0.329
Spiritual/Religious	−0.034	0.339	0.246
Traditional Québec	−0.034	0.436	0.323
Other cultural traditions	0.029	0.403	0.397

### Appendix B.3. Receptivity to Musical Emotions

This subscale was inspired from Schäfer et al. (2013), who identified three core functions of music listening: arousal and mood regulation, self-awareness, and social relatedness. Most items in our version reflect arousal and mood regulation. Adolescents rated how often music elicited specific emotional experiences on a 5-point scale (1 = Never, 5 = Very often). The table below presents the inter-item correlations range and item-total corrected correlations.

**Table A4:** Receptivity to Musical Emotions: Inter-Item Correlation Ranges and Corrected Item–Total Correlations.

Item	Inter-Item *r* (Min)	Inter-Item *r* (Max)	Item–Total *r* (Corrected)
Music puts me in a good mood	0.170	0.669	0.609
Music comforts me	0.293	0.704	0.703
Music reduces my anger	0.107	0.507	0.560
Music makes me feel less lonely	0.287	0.540	0.629
Music gives me pleasure	0.228	0.601	0.662
Music entertains me	0.130	0.518	0.572
Music consoles me	0.303	0.704	0.648
Music makes me nostalgic ^1^	0.107	0.333	0.297
Music makes me feel joyful	0.145	0.669	0.573
Music relieves my boredom	0.112	0.457	0.465

Note. ^1^ The item “*Music makes me nostalgic*” was excluded from the final subscale due to weak correlations with the other items and with the total score.

### Appendix B.4. Musical Affinity Total Score

The composite musical affinity score was calculated by adding the scores from each subscale (range: 2–15). As the composite score (*M* = 7.84; *SD* = 2.27) had limited internal consistency (α = 0.502) and conceptual heterogeneity, it is only used for descriptive purposes. The following table presents the correlations between each subscale and the total musical affinity score.

**Table A5:** Intercorrelations Between Subscales and the Musical Affinity Total Score.

Scale	Musical Experience	Listening Diversity	Emotional Receptivity
Musical Experience	—		
Listening Diversity	0.200 *	—	
Emotional Receptivity	0.173 *	0.447 **	—
Musical Affinity Total	0.635 **	0.827 **	0.673 **

Note. * *p* < 0.05. ** *p* < 0.01.

## References

[B1-behavsci-15-01570] Akkermans J., Schapiro R., Mullensiefen D., Jakubowski K., Shanahan D., Baker D., Busch V., Lothwesen K., Elvers P., Fischinger T., Schlemmer K., Frieler K. (2019). Decoding emotions in expressive music performances: A multi-lab replication and extension study. Cognition and Emotion.

[B2-behavsci-15-01570] American Psychiatric Association (2022). Diagnostic and statistical manual of mental disorders.

[B3-behavsci-15-01570] Ayano G., Demelash S., Gizachew Y., Tsegay L., Alati R. (2023). The global prevalence of attention deficit hyperactivity disorder in children and adolescents: An umbrella review of meta-analyses. Journal of Affective Disorders.

[B4-behavsci-15-01570] Bailey J. A., Penhune V. (2013). The relationship between the age of onset of musical training and rhythm synchronization performance: Validation of sensitive period effects [Original Research]. Frontiers in Neuroscience.

[B5-behavsci-15-01570] Baltazar M., Saarikallio S. (2017). Strategies and mechanisms in musical affect self-regulation: A new model. Musicae Scientiae.

[B6-behavsci-15-01570] Barkley R. A. (2010). Why emotional impulsiveness should be a central feature of ADHD. The ADHD Report.

[B7-behavsci-15-01570] Baweja R., Soutullo C. A., Waxmonsky J. G. (2021). Review of barriers and interventions to promote treatment engagement for pediatric attention deficit hyperactivity disorder care. World Journal of Psychiatry.

[B8-behavsci-15-01570] Berlyne D. E. (1971). Aesthetics and psychobiology.

[B9-behavsci-15-01570] Biasutti M., Habe K. (2023). Teachers’ perspectives on dance improvisation and flow. Research in Dance Education.

[B10-behavsci-15-01570] Biederman J., Faraone S. V., Keenan K., Benjamin J., Krifcher B., Moore C., Sprich-Buckminster S., Ugaglia K., Jellinek M. S., Steingard R. (1992). Further evidence for family-genetic risk factors in attention deficit hyperactivity disorder: Patterns of comorbidity in probands and relatives in psychiatrically and pediatrically referred samples. Archives of General Psychiatry.

[B11-behavsci-15-01570] Biederman J., Faraone S. V., Spencer T. J., Mick E., Monuteaux M. C., Aleardi M. (2006). Functional impairments in adults with self-reports of diagnosed ADHD: A controlled study of 1001 adults in the community. Journal of Clinical Psychiatry.

[B12-behavsci-15-01570] Bonneville-Roussy A., Rentfrow P. J., Xu M. K., Potter J. (2013). Music through the ages: Trends in musical engagement and preferences from adolescence through middle adulthood. Journal of Personality and Social Psychology.

[B13-behavsci-15-01570] Bramley S., Dibben N., Rowe R. (2016). Investigating the influence of music tempo on arousal and behaviour in laboratory virtual roulette. Psychology of Music.

[B15-behavsci-15-01570] Brattico E., Brusa A., Dietz M., Jacobsen T., Fernandes H., Gaggero G., Toiviainen P., Vuust P., Proverbio A. (2025). Beauty and the brain–Investigating the neural and musical attributes of beauty during naturalistic music listening. Neuroscience.

[B14-behavsci-15-01570] Brattico E., Pearce M. (2013). The neuroaesthetics of music. Psychology of Aesthetics, Creativity, and the Arts.

[B16-behavsci-15-01570] Bunford N., Evans S. W., Wymbs F. (2015). ADHD and emotion dysregulation among children and adolescents. Clinical Child and Family Psychology Review.

[B17-behavsci-15-01570] Carrer L. R. (2015). Music and sound in time processing of children with ADHD. Frontiers in Psychiatry.

[B18-behavsci-15-01570] Castro S. L., Lima C. F. (2014). Age and musical expertise influence emotion recognition in music. Music Perception.

[B19-behavsci-15-01570] Cespedes-Guevara J., Eerola T. (2018). Music communicates affects, not basic emotions—A constructionist account of attribution of emotional meanings to music. Frontier Psychology.

[B20-behavsci-15-01570] Chong H. J., Kim H. J., Kim B. (2024). Scoping review on the use of music for emotion regulation. Behavioral Sciences.

[B21-behavsci-15-01570] Christiansen H., Hirsch O., Albrecht B., Chavanon M. L. (2019). Attention-deficit/hyperactivity disorder (ADHD) and emotion regulation over the life span. Current Psychiatry Reports.

[B22-behavsci-15-01570] Dalla Bella S., Farrugia N., Benoit C.-E., Begel V., Verga L., Harding E., Kotz S. A. (2017). BAASTA: Battery for the assessment of auditory sensorimotor and timing abilities. Behavior Research Methods.

[B23-behavsci-15-01570] Dalla Bella S., Foster N. E., Laflamme H., Zagala A., Melissa K., Komeilipoor N., Blais M., Rigoulot S., Kotz S. A. (2024). Mobile version of the battery for the assessment of auditory sensorimotor and timing abilities (BAASTA): Implementation and adult norms. Behavior Research Methods.

[B25-behavsci-15-01570] Demorest S. M., Morrison S. J., Jungbluth D., Beken M. N. (2008). Lost in translation: An enculturation effect in music memory performance. Music Perception.

[B24-behavsci-15-01570] de Oliveira Goes A., Nardi A. E., Quagliato L. A. (2025). Outcomes of music therapy on children and adolescents with attention-deficit/hyperactivity disorder: A systematic review and meta-analysis.

[B26-behavsci-15-01570] Dillman Carpentier F. R., Potter R. F. (2007). Effects of music on physiological arousal: Explorations into tempo and genre. Media Psychology.

[B29-behavsci-15-01570] Eerola T., Ferrer R., Alluri V. (2012). Timbre and affect dimensions: Evidence from affect and similarity ratings and acoustic correlates of isolated instrument sounds. Music Perception: An Interdisciplinary Journal.

[B27-behavsci-15-01570] Eerola T., Vuoskoski J. K. (2011). A comparison of the discrete and dimensional models of emotion in music. Psychology of Music.

[B28-behavsci-15-01570] Eerola T., Vuoskoski J. K. (2013). A review of music and emotion studies: Approaches, emotion models, and stimuli. Music Perception.

[B30-behavsci-15-01570] Ekman P. (1992). An argument for basic emotions. Cognition & Emotion.

[B31-behavsci-15-01570] Fasano M. C., Cabral J., Stevner A., Vuust P., Cantou P., Brattico E., Kringelbach M. L. (2023). The early adolescent brain on music: Analysis of functional dynamics reveals engagement of orbitofrontal cortex reward system. Human Brain Mapping.

[B32-behavsci-15-01570] Fernández-Sotos A., Fernández-Caballero A., Latorre J. M. (2016). Influence of tempo and rhythmic unit in musical emotion regulation. Frontiers in Computational Neuroscience.

[B33-behavsci-15-01570] Ferreri L., Mas-Herrero E., Zatorre R. J., Ripollés P., Gomez-Andres A., Alicart H., Olivé G., Marco-Pallarés J., Antonijoan R. M., Valle M. (2019). Dopamine modulates the reward experiences elicited by music. Proceedings of the National Academy of Sciences of the United States of America.

[B34-behavsci-15-01570] Frankel J. (2006). Reaper *(Version v6.78/win64) [Computer software]*.

[B35-behavsci-15-01570] Freitas C., Manzato E., Burini A., Taylor M. J., Lerch J. P., Anagnostou E. (2018). Neural correlates of familiarity in music listening: A systematic review and a neuroimaging meta-analysis. Frontiers in Neuroscience.

[B36-behavsci-15-01570] Fuentes-Sánchez N., Pastor R., Eerola T., Escrig M. A., Pastor M. C. (2022). Musical preference but not familiarity influences subjective ratings and psychophysiological correlates of music-induced emotions. Personality and Individual Differences.

[B37-behavsci-15-01570] Gabrielsson A., Lindström E., Juslin P. N., Sloboda J. A. (2010). The role of structure in the musical expression of emotions. Handbook of music and emotion: Theory, research, applications.

[B38-behavsci-15-01570] Galván A. (2010). Adolescent development of the reward system. Frontiers in Human Neuroscience.

[B39-behavsci-15-01570] Garrido S., Schubert E. (2011). Individual differences in the enjoyment of negative emotion in music: A literature review and experiment. Music Perception.

[B40-behavsci-15-01570] Ghanizadeh A. (2011). Sensory processing problems in children with ADHD, a systematic review. Psychiatry Investigation.

[B41-behavsci-15-01570] Graziano P. A., Garcia A. (2016). Attention-deficit hyperactivity disorder and children’s emotion dysregulation: A meta-analysis. Clinical Psychology Review.

[B42-behavsci-15-01570] Grewe O., Nagel F., Kopiez R., Altenmüller E. (2007). Listening to music as a re-creative process: Physiological, psychological, and psychoacoustical correlates of chills and strong emotions. Music Perception.

[B43-behavsci-15-01570] Groarke J. M., Hogan M. J. (2019). Listening to self-chosen music regulates induced negative affect for both younger and older adults. PLoS ONE.

[B44-behavsci-15-01570] Groß C., Serrallach B. L., Mohler E., Pousson J. E., Schneider P., Christiner M., Bernhofs V. (2022). Musical performance in adolescents with ADHD, ADD and dyslexia-behavioral and neurophysiological aspects. Brain Sciences.

[B45-behavsci-15-01570] Hartung C. M., Lefler E. K., Abu-Ramadan T. M., Stevens A. E., Serrano J. W., Miller E. A., Shelton C. R. (2025). A Call to Analyze Sex, Gender, and Sexual Orientation in Psychopathology Research: An Illustration with ADHD and Internalizing Symptoms in Emerging Adults. Journal of Psychopathology and Behavioral Assessment.

[B46-behavsci-15-01570] Hirsch O., Chavanon M., Riechmann E., Christiansen H. (2018). Emotional dysregulation is a primary symptom in adult Attention-Deficit/Hyperactivity Disorder (ADHD). Journal of Affective Disorders.

[B47-behavsci-15-01570] Hofbauer L. M., Rodriguez F. S. (2023). Emotional valence perception in music and subjective arousal: Experimental validation of stimuli. International Journal of Psychology.

[B48-behavsci-15-01570] Hove M. J., Gravel N., Spencer R. M. C., Valera E. M. (2017). Finger tapping and pre-attentive sensorimotor timing in adults with ADHD. Experimental Brain Research.

[B49-behavsci-15-01570] Husain G., Thompson W. F., Schellenberg E. G. (2002). Effects of musical tempo and mode on arousal, mood, and spatial abilities. Music Perception.

[B50-behavsci-15-01570] Ipçi M., Inci IzmIr S. B., Türkçapar M. H., Özdel K., Ardiç U. A., Ercan E. S. (2020). Psychiatric comorbidity in the subtypes of ADHD in children and adolescents with ADHD according to DSM-IV. Noropsikiyatri Arsivi.

[B51-behavsci-15-01570] Jakubowski K., Eerola T. (2022). Music evokes fewer but more positive autobiographical memories than emotionally matched sound and word cues. Journal of Applied Research in Memory and Cognition.

[B52-behavsci-15-01570] Juslin P. N., Laukka P. (2004). Expression, perception, and induction of musical emotions: A review and a questionnaire study of everyday listening. Journal of New Music Research.

[B53-behavsci-15-01570] Juslin P. N., Västfjäll D. (2008). Emotional responses to music: The need to consider underlying mechanisms. Behavioral and Brain Sciences.

[B54-behavsci-15-01570] Kahn J. H., Enevold K. C., Feltner-Williams D., Ladd K. (2025). Using music to feel better: Are different emotion-regulation strategies truly distinct?. Psychology of Music.

[B55-behavsci-15-01570] Koelsch S. (2012). Brain and music.

[B56-behavsci-15-01570] Korsmit I. R., Montrey M., Wong-Min A. Y. T., McAdams S. (2023). A comparison of dimensional and discrete models for the representation of perceived and induced affect in response to short musical sounds. Frontiers in Psychology.

[B57-behavsci-15-01570] Krause A. E., North A. C., Hewitt L. Y. (2015). Music-listening in everyday life: Devices and choice. Psychology of Music.

[B58-behavsci-15-01570] Kreutz G., Ott U., Teichmann D., Osawa P., Vaitl D. (2008). Using music to induce emotions: Influences of musical preference and absorption. Psychology of Music.

[B59-behavsci-15-01570] Lachance K.-A., Pelland-Goulet P., Gosselin N. (2025). Listening habits and subjective effects of background music in young adults with and without ADHD. Frontiers in Psychology.

[B60-behavsci-15-01570] Lahdelma I., Eerola T. (2020). Cultural familiarity and musical expertise impact the pleasantness of consonance/dissonance but not its perceived tension. Scientific Reports.

[B61-behavsci-15-01570] Lamont A., Hargreaves D., MacDonald K. V., Hargreaves D. J., Miell D. (2019). Musical preference and social identity in adolescence. Handbook of music, adolescents, and wellbeing.

[B62-behavsci-15-01570] Lapointe M.-C. (2010). L’écoute et la consommation de la musique. Enquête sur les pratiques culturelles au Québec.

[B63-behavsci-15-01570] Larrouy-Maestri P., Morsomme D., Magis D., Poeppel D. (2017). Lay listeners can evaluate the pitch accuracy of operatic voices. Music Perception: An Interdisciplinary Journal.

[B64-behavsci-15-01570] LimeSurvey GmbH (n.d.). LimeSurvey: An open-source survey tool.

[B65-behavsci-15-01570] Liu Y., Liu G., Wei D., Li Q., Yuan G., Wu S., Wang G., Zhao X. (2018). Effects of musical tempo on musicians’ and non-musicians’ emotional experience when listening to music. Frontiers in Psychology.

[B66-behavsci-15-01570] Lorenzo-Quiles O., Soares-Quadros J. F., Abril J. E. (2020). Musical preferences of Brazilian high school students. PLoS ONE.

[B67-behavsci-15-01570] MacGregor C., Müllensiefen D. (2019). The musical emotion discrimination task: A new measure for assessing the ability to discriminate emotions in music. Frontiers in Psychology.

[B68-behavsci-15-01570] Macnamara B. N., Hambrick D. Z., Oswald F. L. (2014). Deliberate practice and performance in music, games, sports, education, and professions: A meta-analysis. Psychological Science.

[B69-behavsci-15-01570] Madjar N., Gazoli R., Manor I., Shoval G. (2020). Contrasting effects of music on reading comprehension in preadolescents with and without ADHD. Psychiatry Research.

[B70-behavsci-15-01570] Magnan S. (2016). Les pratiques culturelles au Québec en 2014: Recueil statistique.

[B72-behavsci-15-01570] Martin-Moratinos M., Bella-Fernandez M., Blasco-Fontecilla H. (2023). Effects of music on attention-deficit/hyperactivity disorder (ADHD) and potential application in serious video games: Systematic review. Journal of Medical Internet Research.

[B73-behavsci-15-01570] Martins M., Pinheiro A. P., Lima C. F. (2021). Does music training improve emotion recognition abilities? A critical review. Emotion Review.

[B71-behavsci-15-01570] Martínez-Vérez V., Gil-Ruíz P., Domínguez-Lloria S. (2024). Interventions through art therapy and music therapy in autism spectrum disorder, ADHD, language disorders, and learning disabilities in pediatric-aged children: A systematic review. Children.

[B74-behavsci-15-01570] McAuley J. D., Jones M. R., Fay R., Popper A. (2010). Tempo and rhythm. Music perception.

[B75-behavsci-15-01570] Mckenna K., Wanni Arachchige Dona S., Gold L., Dew A., Le H. N. (2024). Barriers and enablers of service access and utilization for children and adolescents with attention deficit hyperactivity disorder: A systematic review. Journal of Attention Disorders.

[B78-behavsci-15-01570] Miranda D., Blais-Rochette C., Vaugon K., Osman M., Arias-Valenzuela M. (2013). Towards a cultural-developmental psychology of music in adolescence. Psychology of Music.

[B76-behavsci-15-01570] Miranda D., Claes M. (2009). Music listening, coping, peer affiliation and depression in adolescence. Psychology of Music.

[B77-behavsci-15-01570] Miranda D., Gaudreau P. (2011). Music listening and emotional well-being in adolescence: A person-and variable-oriented study. European Review of Applied Psychology.

[B79-behavsci-15-01570] Morrison S. J., Demorest S. M. (2009). Cultural constraints on music perception and cognition. Progress in Brain Research.

[B80-behavsci-15-01570] Mosing M. A., Madison G., Pedersen N. L., Kuja-Halkola R., Ullén F. (2014). Practice does not make perfect: No causal effect of music practice on music ability. Psychological Science.

[B81-behavsci-15-01570] Mote J. (2011). The effects of tempo and familiarity on children’s affective interpretation of music. Emotion.

[B83-behavsci-15-01570] Musser E. D., Nigg J. T. (2019). Emotion dysregulation across emotion systems in attention deficit/hyperactivity disorder. Journal of Clinical Child & Adolescent Psychology.

[B82-behavsci-15-01570] Müllensiefen D., Gingras B., Musil J., Stewart L. (2014). The musicality of non-musicians: An index for assessing musical sophistication in the general population. PLoS ONE.

[B84-behavsci-15-01570] Park J.-I., Lee I.-H., Lee S.-J., Kwon R.-W., Choo E.-A., Nam H.-W., Lee J.-B. (2023). Effects of music therapy as an alternative treatment on depression in children and adolescents with ADHD by activating serotonin and improving stress coping ability. BMC Complementary Medicine and Therapies.

[B85-behavsci-15-01570] Pearce M. T., Wiggins G. A. (2012). Auditory expectation: The information dynamics of music perception and cognition. Topics in Cognitive Science.

[B86-behavsci-15-01570] Penhune V. B. (2011). Sensitive periods in human development: Evidence from musical training. Cortex.

[B87-behavsci-15-01570] Pereira C. S., Teixeira J., Figueiredo P., Xavier J., Castro S. L., Brattico E. (2011). Music and emotions in the brain: Familiarity matters. PLoS ONE.

[B88-behavsci-15-01570] Peretz I., Gagnon L., Bouchard B. (1998a). Music and emotion: Perceptual determinants, immediacy, and isolation after brain damage. Cognition.

[B89-behavsci-15-01570] Peretz I., Gaudreau D., Bonnel A.-M. (1998b). Exposure effects on music preference and recognition. Memory & Cognition.

[B90-behavsci-15-01570] Porcaro L., Gómez E., Castillo C. (2024). Assessing the impact of music recommendation diversity on listeners: A longitudinal study. ACM Transactions on Recommender Systems.

[B91-behavsci-15-01570] Puyjarinet F., Begel V., Lopez R., Dellacherie D., Dalla Bella S. (2017). Children and adults with attention-deficit/hyperactivity disorder cannot move to the beat. Scientific Reports.

[B92-behavsci-15-01570] Ramos D., Bueno J. L. O., Bigand E. (2011). Manipulating Greek musical modes and tempo affects perceived musical emotion in musicians and nonmusicians. Brazilian Journal of Medical and Biological Research.

[B93-behavsci-15-01570] Rentfrow P. J., Goldberg L. R., Levitin D. J. (2011). The structure of musical preferences: A five-factor model. Journal of Personality and Social Psychology.

[B94-behavsci-15-01570] Rickson D. J. (2006). Instructional and improvisational models of music therapy with adolescents who have attention deficit hyperactivity disorder (ADHD): A comparison of the effects on motor impulsivity. Journal of Music Therapy.

[B95-behavsci-15-01570] Rickson D. J., Watkins W. G. (2003). Music therapy to promote prosocial behaviors in aggressive adolescent boys—A pilot study. Journal of Music Therapy.

[B96-behavsci-15-01570] Romano M., Archambault K., Garel P., Gosselin N. (2024). Music interventions with children, adolescents and emerging adults in mental health settings: A scoping review. Arts & Health.

[B97-behavsci-15-01570] Saarikallio S., Erkkilä J. (2007). The role of music in adolescents’ mood regulation. Psychology of Music.

[B98-behavsci-15-01570] Saarikallio S., Vuoskoski J., Luck G. (2014). Adolescents’ expression and perception of emotion in music reflects their broader abilities of emotional communication. Psychology of Well-Being.

[B99-behavsci-15-01570] Sachs M. E., Damasio A., Habibi A. (2015). The pleasures of sad music: A systematic review. Frontiers in Human Neuroscience.

[B101-behavsci-15-01570] Salimpoor V. N., Benovoy M., Larcher K., Dagher A., Zatorre R. J. (2011). Anatomically distinct dopamine release during anticipation and experience of peak emotion to music. Nature Neuroscience.

[B100-behavsci-15-01570] Salimpoor V. N., Zatorre R. J. (2013). Neural interactions that give rise to musical pleasure. Psychology of Aesthetics, Creativity, and the Arts.

[B102-behavsci-15-01570] Samson S., Dellacherie D., Kolinsky R., Morais J., Peretz I. (2010). La neuropsychologie des émotions musicales. Musique, langage, émotion: Approche neuro-cognitive.

[B103-behavsci-15-01570] Saville P., Kinney C., Heiderscheit A., Himmerich H. (2025). Exploring the intersection of ADHD and music: A systematic review. Behavioral Sciences.

[B104-behavsci-15-01570] Schäfer T., Sedlmeier P., Städtler C., Huron D. (2013). The psychological functions of music listening. Frontiers in Psychology.

[B105-behavsci-15-01570] Schellenberg E. G., Lima C. F. (2024). Music training and nonmusical abilities. Annusl Review of Psychology.

[B106-behavsci-15-01570] Schellenberg E. G., Weiss M. W., Deutsch D. (2013). Music and cognitive abilities. The psychology of music.

[B107-behavsci-15-01570] Scherer K. R., Zentner M. R., Juslin P. N., Sloboda J. A. (2001). Emotional effects of music: Production rules. Music and emotion: Theory and research.

[B108-behavsci-15-01570] Schubert E. (2013). Emotion felt by the listener and expressed by the music: Literature review and theoretical perspectives. Frontiers in Psychology.

[B109-behavsci-15-01570] Shaw P., Stringaris A., Nigg J., Leibenluft E. (2014). Emotion dysregulation in attention deficit hyperactivity disorder. American Journal of Psychiatry.

[B110-behavsci-15-01570] Tan M., Zhou X., Shen L., Li Y., Chen X. (2024). Music’s dual role in emotion regulation: Network analysis of music use, emotion regulation self-efficacy, alexithymia, anxiety, and depression. Depression and Anxiety.

[B111-behavsci-15-01570] Taruffi L., Allen R., Downing J., Heaton P. (2017). Individual differences in music-perceived emotions: The influence of externally oriented thinking. Music Perception: An Interdisciplinary Journal.

[B112-behavsci-15-01570] Tetzner J., Becker M., Maaz K. (2017). Development in multiple areas of life in adolescence: Interrelations between academic achievement, perceived peer acceptance, and self-esteem. International Journal of Behavioral Development.

[B113-behavsci-15-01570] Tillmann B., Bharucha J. J., Bigand E. (2000). Implicit learning of tonality: A self-organizing approach. Psychological Review.

[B114-behavsci-15-01570] Van Den Bosch I., Salimpoor V. N., Zatorre R. J. (2013). Familiarity mediates the relationship between emotional arousal and pleasure during music listening. Frontiers in Human Neuroscience.

[B115-behavsci-15-01570] Vidas D., Nelson N. L., Dingle G. A. (2025). Efficacy of the tuned in music emotion regulation program in international university students. Psychology & Health.

[B116-behavsci-15-01570] Vieillard S., Peretz I., Gosselin N., Khalfa S., Gagnon L., Bouchard B. (2008). Happy, sad, scary and peaceful musical excerpts for research on emotions. Cognition & Emotion.

[B117-behavsci-15-01570] Wåhlstedt C., Thorell L. B., Bohlin G. (2009). Heterogeneity in ADHD: Neuropsychological pathways, comorbidity and symptom domains. Journal of Abnormal Child Psychology.

[B118-behavsci-15-01570] Webster G. D., Weir C. G. (2005). Emotional responses to music: Interactive effects of mode, texture, and tempo. Motivation and Emotion.

[B119-behavsci-15-01570] Williams M. T., Osman M., Kaplan A., Faber S. C. (2024). Barriers to care for mental health conditions in Canada. PLoS Mental Health.

[B120-behavsci-15-01570] Witvliet C. V., Vrana S. R. (2007). Play it again sam: Repeated exposure to emotionally evocative music polarises liking and smiling responses, and influences other affective reports, facial EMG, and heart rate. Cognition and Emotion.

[B121-behavsci-15-01570] Wright N., Moldavsky M., Schneider J., Chakrabarti I., Coates J., Daley D., Kochhar P., Mills J., Sorour W., Sayal K. (2015). Practitioner review: Pathways to care for ADHD—A systematic review of barriers and facilitators. Journal of Child Psychology and Psychiatry.

[B122-behavsci-15-01570] Yik M., Mues C., Sze I. N. L., Kuppens P., Tuerlinckx F., De Roover K., Kwok F. H. C., Schwartz S. H., Abu-Hilal M., Adebayo D. F., Aguilar P., Al-Bahrani M., Anderson M. H., Andrade L., Bratko D., Bushina E., Choi J. W., Cieciuch J., Dru V., Russell J. A. (2023). On the relationship between valence and arousal in samples across the globe. Emotion.

[B123-behavsci-15-01570] Zajonc R. B. (1968). Attitudinal effects of mere exposure. Journal of Personality and Social Psychology.

[B124-behavsci-15-01570] Zatorre R. J., Salimpoor V. N. (2013). From perception to pleasure: Music and its neural substrates. Proceedings of the National Academy of Sciences of the United States of America.

[B125-behavsci-15-01570] Zemestani M., Azadbakht M., Storch E. A. (2023). Preliminary evaluation of music-based emotion-regulation skills to augment CBT for adolescents with ADHD. Musicae Scientiae.

[B126-behavsci-15-01570] Zentner M., Grandjean D., Scherer K. R. (2008). Emotions evoked by the sound of music: Characterization, classification, and measurement. Emotion.

[B127-behavsci-15-01570] Zentner M., Strauss H., Vigl J. (2025). Encapsulating musicality: Development and validation of the Music-Mindedness Questionnaire. Psychology of Aesthetics, Creativity, and the Arts.

[B128-behavsci-15-01570] Zimmermann M. B., Diers K., Strunz L., Scherbaum N., Mette C. (2019). Listening to Mozart improves current mood in adult ADHD—A randomized controlled pilot study. Frontiers in Psychology.

